# Supplementary material for: Comprehensive deep learning model for 3D color holography
Source: Sci Rep. 2022 Feb 15;12:2487. doi: 10.1038/s41598-022-06190-y (PMC8847588; doi:10.1038/s41598-022-06190-y)
Supplement: Supplementary file 1 — Supplementary Information 1. [file 41598_2022_6190_MOESM1_ESM.pdf]

Supplementary material for the article titled: Comprehensive deep learning model for 3D color holography

Alim Yolalmaz[1,2,*] and Emre Yüce[1,2]
[1]*Programmable Photonics Group, Department of Physics, Middle East Technical University, 06800 Ankara, Turkey*
[2]*Micro and Nanotechnology Program, Middle East Technical University, 06800 Ankara, Turkey*
[*]*alim.yolalmaz@metu.edu.tr*
(Dated: January 29, 2022)

Data set generation

Using Eqs. (S1-S3) standing for Fresnel-Kirchhoff diffraction integral, an electric field of light propagates from a hologram plane to a holographic image plane is calculated. The electric field of light at the hologram plane $U_{Hologram}$ is obtained with an incident amplitude of light $A_{incident}$, thickness distribution of the hologram $t_{Hologram}$, refractive index of the hologram material n, and wavelength of light source λ after utilizing Eq. S1. p and q in Eq. S1 are indices of spatially varying amplitude of light at the hologram plane. The light wave from the hologram plane transforms into the image plane with a kernel transformation function G in Eq. S2. The kernel transformation function has light propagation parameters as an observation plane distance from the hologram plane to the image plane d, location of pixels at the hologram plane (x,y), and the image plane (X,Y). a and b are indices at the image plane. Later, we obtain amplitude of light at the image plane U_{Image} with Eq. S3. The intensity of the holographic image I_{Image} is attained with a square of the light amplitude at the image plane U_{Image}.

$$U_{Hologram}(p,q,\lambda) = A_{incident}(p,q,\lambda) * \exp(2\pi j * t_{Hologram}(p,q) * [n(\lambda)-1]/\lambda), \tag{S1}$$

$$G(p,q,a,b,\lambda,d) = \left(\frac{1}{j\lambda d}\right) * \exp\left(\frac{j2\pi d}{\lambda}\right) * \exp\left[j\pi \frac{\left\{(y_{pq}-Y_{ab})^2 + (x_{pq}-X_{ab})^2\right\}}{\lambda d}\right], \tag{S2}$$

$$U_{Image}(a,b,\lambda,d) = \sum_{pq} U_{Hologram}(p,q,\lambda) * G(p,q,a,b,\lambda,d), \tag{S3}$$

Deep learning model

The CHoloNet (see Fig. S1) consists of 10 convolutional neural network (CNN) layers with 64 filters and a filter size of 3-by-3, two max-pooling layers with a size of 2-by-2, a flattening layer, three dense layers with 1600 units, one dense layer with 12800 units, a drop-out layer with a factor of 0.2, a reshaping operation, and a softmax activation function. For tuning weights of the model, we utilize 51200 holographic images at each frequency channel/observation plane and corresponding 8-bit thickness distributions of holograms as a data set. Before tuning weights of the CHoloNet, we convert hologram information to a one-hot vector with eight classes for obtaining better performance with discrete hologram distributions. During training with 90% of all data, the CHoloNet minimizes categorical cross-entropy loss function. The CHoloNet iteratively updates the model's weights and biases using the adaptive moment estimation (Adam) optimizer with a learning rate of 10^{-4} and a batch size of 100 during back-propagation. The model is implemented using TensorFlow, an open-source deep-learning software package. The training and blind testing of the network were performed on a workstation with 32 GB RAM and an NVIDIA Quadro P5000 GPU. The training process lasts less than an hour for 50 epochs. Once the training was completed at each epoch, we test our model with a validation data set, which is 10% of the input data set.

Generalization ability of the CHoloNet

The generalization ability of our model is proved with the data set used for the images in Fig. 3 in the main text. As we mentioned before, 51200 data are responsible for developing our neural network. During the data set generation, different hologram structures

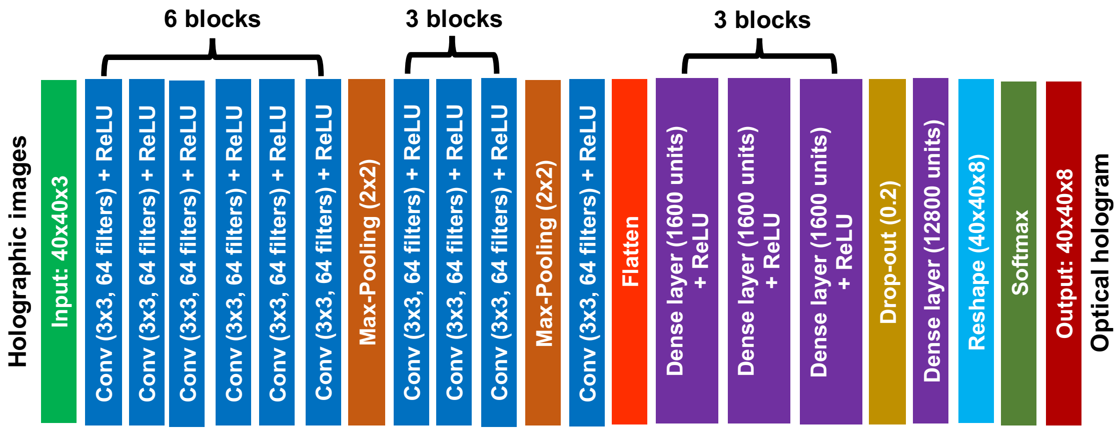

FIG. S1. The CHoloNet architecture. Each color represents a different data operation. The model takes holographic images with a size of 40-by-40-by-3, performs feature extractions through several CNN layers, and correlates holographic images to holograms with a size of 40-by-40-by-8 by using detected features.

are obtained to result in holographic images of the letters. Different holograms enable us to acquire different intensity distributions of the light at the image plane. Our model learns the relation between the holographic images and the holograms. Generalization of the model seems inherently possible with the model due to different intensity images within the data set. To prove our claim, we firstly reconstructed all the holograms which generate intensity images within the data set with our model. Then we calculate correlation values between the ground-truth and the neural network-based holograms. As seen in Fig. S2 our model reconstructs the holograms with correlation coefficients ranging between 95% and 100%, and we encounter a mean correlation of 99.7%. As a result, we conclude that our model reconstructs holograms not only for imaging letters but also for modulating incident light to obtain different intensity distributions at the output plane.

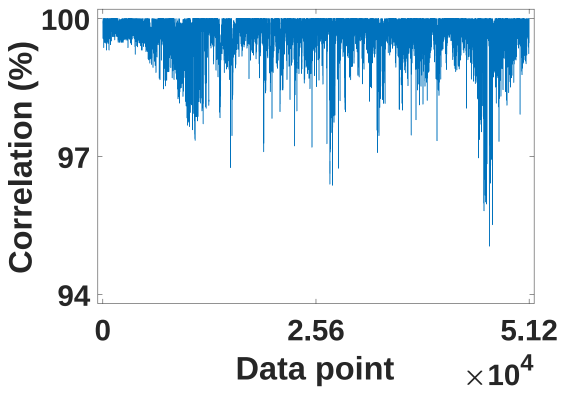

FIG. S2. Correlation coefficients of all reconstructed holograms with the CHoloNet for the intensity images within the data set.
